# Caregivers’ Experiences Regarding Training and Support in the Post-Acute Home Health-Care Setting

**DOI:** 10.1177/2374373519869156

**Published:** 2019-08-21

**Authors:** Jo-Ana D Chase, David Russell, Meridith Rice, Carmen Abbott, Kathryn H Bowles, David R Mehr

**Affiliations:** 1 Sinclair School of Nursing, 16125 University of Missouri, Columbia, MO, USA; 2 Department of Sociology, 1801 Appalachian State University, Boone, NC, USA; 3 Center for Home Care Policy & Research, Visiting Nurse Service of New York, New York, NY, USA; 4 Department of Physical Therapy, 16125 University of Missouri, Columbia, MO, USA; 5 School of Nursing, 16142 University of Pennsylvania, Philadelphia, PA, USA; 6 Department of Family and Community Medicine, 12271 University of Missouri School of Medicine, Columbia, MO, USA

**Keywords:** older adults, physical functioning, hospitalization, qualitative research, home health, post-acute

## Abstract

**Background:**

Post-acute home health-care (HHC) services provide a unique opportunity to train and support family caregivers of older adults returning home after a hospitalization. To enhance family-focused training and support strategies, we must first understand caregivers’ experiences.

**Objective:**

To explore caregivers’ experiences regarding training and support for managing older adults’ physical functioning (PF) needs in the post-acute HHC setting.

**Method:**

We conducted a qualitative descriptive study using semi-structured telephone interviews of 20 family caregivers. Interviews were recorded, transcribed, and analyzed using conventional content analysis.

**Results:**

We identified the following primary categories: facilitators to learning (eg, past experience, learning methods), barriers to learning (eg, learning on their own, communication, timing/logistics, preferred information and timing of information delivery), and interactions with HHC providers (eg, positive/negative interactions, provider training and knowledge).

**Conclusion:**

Caregivers were responsive to learning strategies to manage older adults’ PF needs and, importantly, voiced ideas to improve family-focused training and support. HHC providers can use these findings to tailor training and support of family caregivers in the post-acute HHC setting.

## Introduction

Family caregivers (henceforth, “caregivers”) include any relative, partner, or friend with whom the care recipient is closely familiar (1,2). Almost all caregivers of older adults provide assistance with physical functioning (PF) needs, such as mobility and self-care (3,4). After a hospitalization, older adults may have increased PF needs for which caregivers are unprepared, and information may be needed regarding the older adults’ medical care and how caregivers’ roles can impact patient outcomes (5). Consequently, national organizations have called for better caregiver training and support during hospital-to-home transitions to enhance preparedness and promote family-focused care (3,6-8).

Although current policies exist to support caregiver involvement during the hospital discharge process, these policies have been inconsistently implemented (1,5). For example, the Caregiver Advise, Record, and Enable (CARE) Act requires hospitals to identify, inform, and educate caregivers during the hospital discharge process; however, the CARE Act is yet to be passed in all states, and it varies in its operationalization from state to state (1). Evidence further suggests that caregivers do not receive adequate information regarding their roles and patients’ expected care goals during the discharge process (5,9-12). Even when there has been good discharge teaching, questions inevitably arise after discharge (13).

Thus, caregivers, who often assume the primary responsibility for managing and coordinating post-acute services for care recipients when they return home, are frequently unprepared and forced to handle these tasks alone (9). Poor preparation to manage an older adult’s care when they return home after a hospitalization (hospital-to-home transition) places caregivers at risk of greater stress, burden, and poorer quality of life (14,15). Therefore, additional caregiver training and support is frequently needed once the older adult returns home.

Post-acute skilled home health care (HHC) provides an additional opportunity for caregivers to receive training and support. Many older adults experiencing a hospital-to-home transition receive skilled HHC (a Medicare part A benefit), making up about one-third of the HHC population (16). Patients and their caregivers may have consistent interaction with HHC staff through multiple visits from nurses, physical or occupational therapists, and home health aides. As caregivers perceive post-acute HHC services as a supportive and beneficial resource (17), their interactions with HHC providers could facilitate caregivers’ learning. Learning in this context is defined as the acquisition of skills and resources to assist caregivers in adapting to changes in their roles and responsibilities (18). HHC providers, especially nurses, can empower caregivers by engaging them in planning and decision-making, providing education, and acknowledging the importance of their caregiver roles (19). Thus, post-acute HHC services can act as a bridge, linking and reinforcing discharge education from the hospital and addressing educational gaps with ongoing assessment, training, and support at home.

However, lack of focus on the post-acute setting (19-23) has limited research regarding how well HHC currently addresses caregivers’ training and support needs. One study highlighted differences in care goals among caregivers and HHC provider (20); moreover, caregivers perceive important gaps in communication and HHC services that affect their ability to manage older adults’ care needs (21-23). To date, however, there has been no examination of caregivers’ experiences regarding training and support specific to the post-acute HHC setting. This information is critical to identifying potential targets for future, family-focused interventions to effectively train and support caregivers in the post-acute HHC setting. Understanding caregivers’ perceived facilitators and barriers to learning how to assist with older adults’ PF needs as well as eliciting their descriptions of interactions with HHC providers is an important first step toward identifying gaps in training and support needs. Thus, our study aim was to explore caregivers’ experiences regarding training and support for managing older adults’ PF needs in the post-acute HHC setting. Our research questions were:

1. What are caregivers’ perceived barriers and facilitators for learning how to assist with older adults’ PF needs in the post-acute HHC setting?
2. How do caregivers describe their interactions with HHC providers when receiving training and support to assist with older adults’ PF needs?

## Method

We addressed our study aims through a qualitative descriptive approach (24-26). This report is part of a larger investigation into caregivers’ experiences managing PF needs in the post-acute HHC setting. We previously reported on caregivers’ perceptions and experiences managing older adults’ complex PF care needs following transition from hospital to home (17). Although both reports concern caregivers of patients receiving post-acute HHC, our focus here is specific to caregivers’ experiences receiving training and support in this setting.

### Sampling and Recruitment

We used maximum variation sampling to obtain a racially/ethnically diverse sample of caregivers of older adults who received HHC services following hospitalization (27). The research team collaborated with a large, urban not-for-profit HHC agency located in the northeastern United States. Not-for-profit agencies bill payers for services as do for-profit agencies but are owned by a not-for-profit corporation instead of a profit-making business. Not-for-profit corporations are those that are formed for reasons other than financial gain and that provide charitable benefits to the community (28). We first consecutively identified patients who (1) 6 months prior to the study start were discharged home from the hospital and received HHC following hospitalization, (2) were 65 years or older, and (3) had a caregiver to assist with activities of daily living and/or instrumental activities of daily living. A research assistant then contacted caregivers of eligible patients by phone to explain the study to them. If caregivers were interested in participating, they were subsequently screened for cognitive impairment at this initial recruitment call by the research assistant. Eligible caregivers spoke English and scored ≥3 on the Callahan Six-Item Screener for Cognitive Impairment (29). No caregivers had scores indicating cognitive impairment. Thirty-four caregivers were contacted by the research assistant for recruitment into the study. Five declined to participate due to lack of interest. One declined due to recent passing of the patient. Twenty-eight caregivers were subsequently called for telephone interviews; however, 8 did not return our calls. Twenty caregivers consented to participate and were successfully interviewed. Institutional review board approval was obtained from (Visiting Nurse Service of New York) and (The University of Missouri).

### Data Collection

Caregivers were interviewed by telephone. Two team members (JC, MR) performed the semi-structured interviews, asking sequential primary questions with probing questions to elicit additional depth. The data for this paper and our earlier publication were obtained in the same interviews; however, specific questions pertaining to this article are illustrated in Table 1. Interviews were recorded and transcribed verbatim. We confirmed transcription accuracy line-by-line with the audio file. Caregiver demographics (eg, age, gender, race/ethnicity, and education), self-reported health, relationship with the older adult, years of caregiving experience, and living arrangements (eg, lives with older adult, yes/no) were collected. Caregiver location, proximity of patient, or time of day of interviews were not collected.

**Table 1.** Sample Caregiver Questions.

Sample Questions and Probes
Could you describe any teaching, training, or support to help X recover his/her mobility or activity levels provided by home care? Probe: How did the home care provider deliver the teaching, training, support? Probe: Was this teaching, training, or support helpful? If so, how was it helpful? If not, why was it not helpful? Probe: How could the teaching, training, or support have been more helpful?
I would like you to think about the first time the home care provider visited X. Can you describe to me what happened during the first home care visit after X returned home from the hospital? How soon after you got home was the first home care visit? Was the home care provider a nurse? Physical therapist? Other? Was the home care provider knowledgeable?
Can you describe any discussions about the patient’s goals of care (eg, functional recovery)?
Can you describe other topics covered by the home care provider? Did you ask any questions? And if so, what questions did you ask? If not, why didn’t you ask any questions?

### Qualitative Content Analysis

Caregiver characteristics were summarized using descriptive statistics. The unit of analysis was the transcriptions of caregiver interviews (30). To analyze the interview data, we applied conventional content analysis, a systematic method of coding text and identifying patterns in qualitative data (31,32). Two researchers independently read each full interview, then reread them line-by-line to develop in vivo codes using Dedoose software (33). We developed subcategories of similarly grouped codes across all interviews from which a set of main categories were identified (32,34).

We used multiple strategies to ensure trustworthiness and analytic rigor (35). We maintained an audit trail of all coding decisions and analysis. Two researchers independently coded each interview and reviewed coding results together (investigator triangulation). Inter-rater reliability was assessed for frequently applied codes with excellent agreement (κ scores ranged between 0.82 and 0.84). For member checking, our findings were discussed with a patient advisory board, consisting of patients and caregivers, who agreed with our analyses.

## Results

Caregiver characteristics are shown in Table 2. Caregivers were mostly female (70%). Thirty-five percent of the sample were white, 40% were black, and 25% were Hispanic-Latino. Caregivers’ mean age was 58 (standard deviation [SD] = 13). Most caregivers were caring for a parent (65%). On average, caregivers had been caring for their family member for 14 years (SD = 13). Overall, caregiver participants were well educated (70% had at least some college education or were college graduates), and 50% of the sample were employed. Forty-five percent reported their health as excellent to very good, and 55% reported their health as good to fair. Average interview time was 19 minutes (SD = 8, range 9:06-42:14).

**Table 2.** Caregiver Characteristics.^a^

Variable	n or Mean (SD)
Caregiver characteristics	
Mean age (SD)	58 (13)
Female	14
Married	7
Race/ethnicity	
White	7
Black	8
Hispanic/Latino ethnicity	5
Education	
At least some college	14
High school or GED	6
Employment status	
Retired	8
Employed	10
Unemployed	2
Self-reported health	
Excellent to very good	9
Good to fair	11
Caregiving characteristics	
Mean years providing care (SD)	14 (13)
Care recipient	
Parent	13
Spouse	1
Sibling	3
Grandparent	1
Friend	2
Lives with care recipient	10

Abbreviations: SD, standard deviation; GED, general education development.

^a^ N = 20.

Table 3 contains the 3 primary categories with subcategories and exemplar quotes of caregivers’ experiences regarding training and support in the post-acute HHC setting. We assigned pseudonyms for quotes and provided age and relationship with the care recipient.

**Table 3.** Summary of Categories and Sub-Categories With Exemplar Quotes.

Category 1a: Facilitators to Learning How to Manage PF Needs
“*Past experience*” included previous family caregiving and work experiences which helped prepare caregivers to manage the PF needs of older adults after hospitalization. “…sickness been around in the family for a long time. So guess that’s where [my experience] all come from, right.?” (Steven, 60, husband]. “When I was going through CNA training, watching the visiting nurse services have done for both my mother and grandmother, that’s kind of help me prepare for any time, you know, she has to go in the hospital and she does come home” (Ellen, 29, grand-daughter). “*Asking questions*” was an important means of information-seeking for caregivers. “If I have questions, I’ll ask them when they’re there. Should my mom be doing this or shouldn’t she be doing that? I’ll question them, like when the therapist is here with my mom. How long is she supposed to do therapy? How long is she supposed to do it for? How long is she supposed to stand for? Things like that. Yeah, I did ask questions just to make sure because she’ll know I know, so just because they’re not here doesn’t mean that she [doesn’t need to do it]” (Jane, 46, daughter). “*They showed me how*” describes caregivers’ observations of HHC providers. “I just followed suit. They didn’t give me any formal training or anything. I just watched what they did and then just continued doing it” (Mike, 53, Son). “*Written Instructions*” were instructions left behind by HHC providers that helped caregivers monitor patients’ recoveries and comply with exercise plans. “The [physical] therapist left a 8 by 10 paper, instructions on how to exercise her when she not here.” [Steven, 60, husband]
Category 1b: Barriers to Learning How to Manage PF Needs
“*I learn on my own*” describes caregivers’ lack of receiving formal training to manage the PF needs of older adults in the post-acute setting. “Well, I read, you know, by my reading, but [nobody] comes here and train me how to do this, nobody. I learn on my own” (Kate, 62, daughter). “*The most important things*” describes caregivers’ preference for the types of information that would be helpful to manage PF in the post-acute setting “I would say the most important things I need to know is, you know, is she progressing in her treatment. Is, you know, are her, are the long-term health goals being met, and whether or not there’s any, you know, changes that the nurse feels may be necessary to ensure that, you know, she, her, her quality of life stays the same or improves” (Roger, 49, Son). “I think that would have helped if they had asked me more…there were some things that I didn’t think were a big deal” (Cindy, 54, daughter). “*I got the run-around*” described issues with communications, including communication between providers and caregivers/patients and between providers themselves “It’s kind of, you know, difficult. There’s a lot of folks involved in her care, I guess, and because they’re all, you know, not necessarily communicating with one another, you tend to have to repeat yourself a lot. (My mom) gets frustrated that way.” [Roger, 49, Son]
Category 1b: Barriers to Learning How to Manage PF Needs
*Issues with timing and logistics* describes the timing of interactions with HHC providers, and disruption and length of services which created challenges in managing the PF needs of older adults. “If you’re in the hospital, when you get home, they don’t usually come the same day…they usually don’t come until the next day” (Helen, 68, daughter). “I think it is really important that there’s extra information given, like a couple weeks is not enough. I think they should be more informative if they only have that couple of weeks, they should have more information to give you, and that’s what they did on the phone program. They were giving me more information, asking me different questions than they did in the house. So I think that would have helped more, if they had asked me more so there were some things that I didn’t think were a big deal. They did, and they asked me more questions about afterwards, so that helped a lot” (Cindy, 54, daughter). *“Given to me sooner*” describes caregivers’ preferences for the timing and delivery of training and support. “So, if something could be improved, I would think, if possible, to have that visiting nurse come the day you come home. That would be my biggest request to everybody, that they should improve one, so that when you get home, that person comes and checks [up] and sees you” (Helen, 68, daughter).
Category 2: Interactions with HHC Providers When Receiving Training and Support
“*Good match*” described characteristics that caregivers perceived as positive among HHC providers “I think the perfect [home health aide] will be a person who’s calm and caring and showing that she’s there mainly to help her, assist her and, you know, because, they are alive and they do have a mind” (Tara, 71, daughter). “*They didn’t interact*” described characteristics caregivers perceived as negative among HHC providers “Oh, well, first and foremost, you know, conversation…I told the girl, ‘Can you go in and talk to her?’ Do you know how heartbroken it is to walk in and just see your mother just sitting there staring down the foyer, doing nothing. That’s what I told the girl…Go in and sit with her…some kind of conversation.’ I, my mother didn’t seem to have luck with aides and nurses, she really didn’t. Like some, I know some people that have them and they’re interacting with them. I don’t know they just, they didn’t interact” (Donna, 54, daughter). “*Wish s/he knew more*”: describes the types of information and training that caregivers would prefer among HHC providers. “I did ask [the nurse] if I was using the machine correctly and if I should be doing anything else because I wasn’t, you know, told anything from the hospital. So was there anything I should do? There was not even a plan, no paperwork given to me that I found out that you’re supposed to get instructions. I didn’t know that. So she said she would check with the doctor and that was it really. She didn’t have any information on the case” (Iris, 58, sister). “It’s just that I feel that [HHAs] need some kind of, you know, classes, they should have more of an open mind and a little bit more compassion…know how to handle people, especially, seniors that live alone and, you know, disabilities” (Tara, 71, daughter).

Abbreviations: PF, physical functioning; HHC, home health care.

### Category 1a: Facilitators to Learning How to Manage PF Needs

Caregivers reported diverse facilitators for learning how to manage PF needs of older adults after hospitalization, which are represented by the subcategories past experience, asking questions, they showed me how, and written instructions. Past experience included previous family caregiving experiences which helped prepare caregivers to manage the PF needs of older adults after hospitalization. For example, one caregiver said: “I’ve been doing this since I was 11 years old, so knowing my mom, I know what I have to do” (Jane, 46, daughter). Past experiences also included health-care work experience for some caregivers.

Asking questions of health-care providers was an important means of actively seeking information for caregivers. One caregiver stated, “I’m the person who asks a lot of question, I have a little book where I write everything down” (Patricia, 60, daughter). Caregivers’ questions included topics such as how to use specific equipment for mobility, and what limitations were needed on older adults’ activities.

They showed me how describes caregivers’ observations of HHC providers. Several caregivers observed physical therapists and nurses demonstrating tasks during visits, which many caregivers felt was the extent of their training. As one caregiver described, “watching what’s going on around me has prepared me for helping to take care of my grandma” (Ellen, 29, granddaughter).

Caregivers reported that *written instructions* left by HHC providers facilitated learning. These instructions helped caregivers monitor older adults’ recoveries and comply with exercise plans. Written materials typically included illustrations (“picture examples”) and served as “reminders” for how and when to perform rehabilitation exercises.

### Category 1b: Barriers to Learning How to Manage PF Needs

Caregivers described various barriers to learning how to manage the PF needs of older adults after hospitalization, which are represented by the subcategories I learn on my own; the most important things; I got the run-around; issues with timing and logistics; and given to me sooner. I learn on my own describes caregivers’ lack of receiving formal training to manage the PF needs of older adults in the post-acute setting. The absence of training for caregivers created a steep learning curve. As one caregiver described: “Well, at the beginning, it was difficult because I had to kind of train myself and educate myself” (Beth, 58, daughter). The lack of formal training received by caregivers also left them with gaps in knowledge such as “the signs to look for before…a crisis” (Ned, 39, son), or needing more information on how to use different medical equipment.

The more important things reflects caregivers’ preferences for the types of information that would be helpful to manage PF in the post-acute setting. One caregiver stated she would have liked more information “on what to do when I got her up and had to give her medication, and her physical activity” (Cindy, 54, daughter). Other caregivers wanted more information on goals and expectations for care and whether or not the older adult was “progressing in his/her treatment.” Some caregivers felt HHC providers need to ask more questions of caregivers to assess for gaps in comprehension and skills.

*I got the run-around* describes issues with communications, including communication between providers and caregivers/patients and between providers themselves. Reasons for this feeling included difficulty accessing providers (eg, “it’s just being on the phones to get through, to wait, you gotta hang on, press buttons, very difficult.” [Gail, 68, daughter]), or receiving no response (eg, “Nobody answers. I’ve faxed, the doctor has faxed it, I have faxed it, nobody, no answer whatsoever…It’s very hard.” [Kate, 62, daughter]). Additionally, caregivers noted lack of provider-to-provider communication resulting in caregivers and patients having to repeat themselves.

Issues with timing and logistics describes the timing of interactions with HHC providers and disruption and length of services which created challenges in managing the PF needs of older adults. Several caregivers would have liked to have a nursing evaluation earlier in the post-acute period. Other caregivers commented on the disruption of services that occurred due to the hospitalization, affecting how they were able to manage the older adult’s care. Caregivers had to struggle to coordinate resumption of HHC services to manage the older adult’s PF needs. Caregivers also felt as though services were “too short” and that HHC providers “should be more informative” regarding goals of care and descriptions of services.

Caregivers described concerns for timing and delivery of training and support, reflected in the subcategory given to me sooner. Regarding her mother’s goals of care, one caregiver stated:

I think if it was given to me sooner, if there was more knowledge by the [home health care] service, it would have been better because I could have gotten her up and maybe the swelling or what have you, whatever they call it that the water built up, would have been out and she wouldn’t have been laying around…But I think if I got the information sooner it would have helped (Cindy, 54, daughter).

Additionally, several caregivers felt that early visits, such as the day of returning home, would better prepare them to manage PF needs.

### Category 2: Interactions With HHC Providers When Receiving Training and Support

Overall, caregivers felt that HHC services were beneficial in improving the older adult’s PF status and preventing hospitalization. Caregivers’ perceptions of interactions with HHC providers included their observations on how HHC providers worked with them and the older adult and their judgment of HHC providers’ knowledge base and training. Caregivers’ positive perceptions of interactions with HHC providers promoted communication and trust, whereas negative perceptions inhibited the development of relationships that were built upon these characteristics. Caregivers described interactions with HHC providers in a variety of ways represented by subcategories of *good match*; *they didn’t interact*; *and wished s/he knew more.*

Positive experiences were described as *a good match.* The HHC provider characteristics that constituted a good match included being “knowledgeable,” “patient,” “attentive,” “caring,” “friendly,” “honest,” and “compassionate.” Caregivers described positive relationships between the older adult and HHC providers as ones involving “camaraderie” and “respect.” Caregivers often described the older care recipient as “stubborn” or “grumpy,” and they preferred HHC providers (specifically home health aides) who understood how to communicate with older adults and involved both the older adult and themselves in conversation and training.

Caregivers also described poor interactions with HHC providers, with one caregiver summarizing these negative interactions as *they just didn’t interact.* Negative characteristics included being “not attentive,” “not taking care of [the older adults’] needs,” and being “irresponsible.” Lack of training among home health aides was another negative characteristic that caregivers felt contributed to poor interactions. Multiple caregivers stated that some home health aides were not “trained properly” or “just get a little training.”

Wish she/he knew more describes the types of information and training that caregivers would prefer among HHC providers which could enhance training and support interactions. Several caregivers noted that some HHC nurses “didn’t have any information” on the patient and wished the nurses “knew more about [the patient].” However, another caregiver stated that the HHC nurses did work hard to find information for them. Caregivers also highlighted the need for HHC providers to be familiar with or trained in caring for older adults, especially for older adults with significant functional deficits.

## Discussion

Post-acute HHC services can serve as an important resource for caregivers managing the PF needs of older adults who experience hospital-to-home transitions. These caregivers need actionable information, continuity of care across clinical settings, and empathy in communication (11). Our research has practical applications for HHC providers and agencies seeking to improve training and support of caregivers of older adults in the post-acute setting.

Overall, caregivers in our sample were inquisitive and proactive learners. Feeling prepared to manage the patient’s anticipated care needs is important for both caregivers and patients experiencing hospital-to-home transitions (11). Although many caregiver participants had existing knowledge and experience of the care recipients’ PF needs, for some, declines in PF after a hospitalization presented new challenges. Thus, caregivers’ training needs are dynamic, and ongoing assessments are necessary to adapt and tailor educational interventions over time to provide preferred information. Caregivers in our study highlighted the types of information that were most helpful for them, including the provider’s expectations for the older adult’s clinical progression and quality of life. The HHC providers should first assess caregivers’ learning needs and preferences to tailor training strategies (eg, verbal, observation, and written). Educational interventions should be tailored to each individual patient’s post-acute care needs and family and community resources to produce actionable information (11,12,36).

Caregivers also identified important barriers to learning how to manage PF needs, many of which were related to the logistics of post-acute care. For example, caregivers were often confused about whom to contact once the older adult returned home. Additionally, the timing of HHC services was a challenge, with some caregivers asking for a nursing visit the day they returned home from the hospital. Other caregivers discussed the brevity and fragmentation of services. Logistical and communication issues in the HHC setting increase caregivers’ sense of isolation and burden (37,38). HHC nurses could collaborate with caregivers to coordinate care and identify clear points of contact for both HHC services and follow-up care to improve care continuity. Furthermore, incorporating interventions after discharge from HHC services, such as periodic telephone follow-up or telehealth, could be used to deliver ongoing caregiver training and support (39,40).

Part of fostering a supportive learning environment in the post-acute HHC setting is establishing a professional and trusting relationship among HHC providers and family caregivers (21). Descriptions of positive encounters with HHC providers highlighted professional training and demeanor. These findings are consistent with research from Byrne and colleagues in which caregivers felt more comfortable among HHC providers they perceived as “adept” versus “inept” in the clinical roles. HHC agencies may address caregivers’ concerns by training home health aides in motivational interviewing and health coaching techniques for health promotion, as these strategies have been beneficial among patients with chronic illness (41). Furthermore, compassionate communication and empathy are essential to successful care transitions for older adults and their caregivers (11). To improve HHC providers’ sensitivity to the unique experiences of caregiving of older adults, HHC agencies should consider offering education on aging- and family-focused care for all HHC providers (20,42). Indeed, national initiatives are needed to promote this type of training across all provider disciplines, given the growing number of adults aged 65 and older who comprise the majority of HHC patients (43) and the well-documented needs of their caregivers (6,8,3).

Also contributing to caregivers’ perceptions of HHC provider “ineptness” was frustration with nurses who were unfamiliar with the older adults’ case. Past research has highlighted HHC nurses’ own awareness of the information disconnect during care transitions (44-46) and the subsequent impact on their practice(47,48). Agency and systems-level interventions, including those that facilitate seamless communication between hospital and HHC agency staff, are needed to ensure that nurses are well informed of patient cases prior to visits. Evidence-based interventions to improve communication between providers should include the use of health information technology, such as electronic medical records, and efficient integration of clinical records to improve information management across care settings (49). Assessments of caregivers that account for caregivers’ experiences and learning preferences and incorporate evaluation of training and support gaps could be captured during the hospital discharge process and shared with post-acute providers including HHC agencies. Health information technology may also facilitate HHC nurses’ ability to coordinate care, enhance care continuity, track training and support efforts for caregivers and patients, and communicate with other providers (11,45).

### Strengths and Limitations

A primary strength of this study is the use of a subset of caregivers—those caring for older adults during an important care transition—to examine their experiences and recommendations for improving training and support in the post-acute HHC setting. Importantly, the qualitative nature of the study elicited caregivers’ voices to describe their preferences to address training and support gaps in the post-acute HHC setting. Another study strength was the racial/ethnic diversity of the sample, which reflects the current racial/ethnic demographics of family caregivers across the United States (3,7).

This study had limitations, including a small sample size although we achieved data saturation. It is possible that the caregivers who declined study participation or who did not return our calls may have had different experiences from those we interviewed, limiting the reliability and transferability of our study findings. Our sample was recruited from a single, large HHC agency in the northeast, which serves a largely urban population; thus, study findings may not be generalizable to populations in other areas of the nation, especially those served by smaller HHC agencies in rural areas. The study scope was specific to managing the PF needs of older adults in the post-acute HHC setting, given the large proportion of caregivers who assist with these activities (3,4). However, caregivers are involved in a broad range of care activities, including medical/nursing tasks (3,9).

## Conclusion

Caregivers face challenges in learning how to manage the PF needs of older adults during hospital-to-home transitions. Post-acute HHC services can be a critical resource for these caregivers. Our study findings highlight specific barriers and facilitators to learning, and factors impacting collaborative interactions among caregivers and HHC providers. These findings may be used to inform family-focused strategies for caregiver training and support. Additionally, HHC agencies should educate providers in aging- and family-focused principles and facilitate communication of clinical information across disciplines and care settings. Further research should incorporate a wider examination of strategies to enhance caregiver preparedness for more diverse roles and care activities.
